# Treatment of non-small-cell lung cancer: a perspective on the recent advances and the experience with gefitinib

**DOI:** 10.1038/sj.bjc.6602062

**Published:** 2004-08-31

**Authors:** A Onn, M Tsuboi, N Thatcher

**Affiliations:** 1 Department of Thoracic/Head and Neck Medical Oncology, University of Texas MD Anderson Cancer Center, Box 403, 1515 Holcombe Boulevard, Houston, TX 77030-4009, USA; 2 Department of Surgery, Tokyo Medical University, Tokyo, Japan; 3 Department of Medical Oncology, Christie Hospital NHS Trust, Manchester, UK

**Keywords:** gefitinib (‘Iressa’), EGFR-TKI, NSCLC, chemotherapy

## Abstract

Worldwide, non-small-cell lung cancer (NSCLC) is a leading cause of cancer-related mortality and, until screening detects early disease, treatment for the majority of patients will consist of radiation therapy, chemotherapy or combinations thereof. Modern mono and doublet chemotherapy regimens have translated into modest increases in life expectancy and improved quality of life, but at the expense of systemic and pulmonary adverse events (AEs). There is a great unmet need to provide effective therapy for advanced NSCLC that does not have the toxicity burden of conventional chemotherapy and radiotherapy. Novel drugs that inhibit a range of growth factor receptors, such as the epidermal growth factor receptor tyrosine kinase inhibitors gefitinib (‘Iressa’) and erlotinib (‘Tarceva’) or the monoclonal antibody cetuximab (‘Erbitux’), have recently been evaluated. Having demonstrated antitumour activity and rapid symptom improvement in pretreated patients with advanced NSCLC, gefitinib was approved in the USA, Japan and other countries. Gefitinib is well tolerated with a low incidence of grade 3/4 AEs. Interstitial lung disease has been reported in a small number of patients receiving gefitinib, although this may be attributed to other treatments and conditions. Nevertheless, although the use of novel treatments requires vigilance for unexpected AEs such as pulmonary toxicity, in this area of high unmet clinical need, the benefits outweigh the risks in patients for whom no other proven effective treatment exists.

## NON-SMALL-CELL LUNG CANCER (NSCLC)

Lung cancer is one of the most common malignancies in developed countries and accounts for millions of deaths worldwide. In 2000, the annual incidence of NSCLC, which comprises 80% of all lung cancer cases, was 991 089 and the worldwide mortality was 882 495 (Ferlay *et al*, 2001).

At diagnosis, patients with NSCLC can be divided into three groups, reflecting disease extent and treatment approach. The first group, patients with stage I and II disease, has the best prognosis and surgery is the treatment of choice; however, patients with inoperable disease might be considered for radiation therapy with curative intent. Careful preoperative assessment of the patient's overall pulmonary reserve is essential in considering surgery. The second group of patients (stage III) includes those with locally or regionally advanced NSCLC. These patients are often treated with combined modality treatment, that is, various combinations of chemotherapy, radiation therapy and surgery. Patients in the final group (stage IV) have advanced disease with distant metastases and can be treated with radiation therapy and/or chemotherapy.

Unfortunately, the prognosis of patients with advanced NSCLC remains poor. Systemic chemotherapy prolongs survival compared with best supportive care alone, as demonstrated in patients treated with platinum-based regimens (Non-small Cell Lung Cancer Collaborative Group, 1995). However, chemotherapeutic regimens are associated with systemic (Table 1

**Table 1:** Common AEs associated with cancer therapy (Schiller *et al*, 2002)

**Treatment**	**Drug dose and regimen**	**Highest grade (all AEs, %)**	**Most common grade 3/4 AEs**
Cisplatin and paclitaxel	Paclitaxel 135 mg m^−2^ over 24-h period	Grade 3 (19)	Absolute neutrophil count
*n*=300	on day 1;	Grade 4 (68)	Nausea
	Cisplatin 75 mg m^−2^ on day 2;	Grade 5 (5)	Vomiting
	Repeat cycle every 21 days		Febrile neutropenia
			
Cisplatin and gemcitabine	Gemcitabine 1000 mg m^−2^ on days 1, 8,	Grade 3 (21)	Absolute neutrophil count
*n*=293	15;	Grade 4 (68)	Platelet count
	Cisplatin 100 mg m^−2^ on day 1;	Grade 5 (4)	Nausea
	Repeat cycle every 28 days		Vomiting
			
Cisplatin and docetaxel	Docetaxel 75 mg m^−2^ on day 1;	Grade 3 (23)	Absolute neutrophil count
*n*=287	Cisplatin 75 mg m^−2^ on day 1;	Grade 4 (61)	Nausea
	Repeat cycle every 21 days	Grade 5 (6)	Vomiting
			Weakness
			
Carboplatin and paclitaxel	Paclitaxel 225 mg m^−2^ over 3-h period	Grade 3 (28)	Absolute neutrophil count
*n*=293	on day 1;	Grade 4 (53)	Weakness
	Carboplatin, AUC 6.0 mg ml^−1^ min^−1^ on	Grade 5 (4)	Platelet count
	day 1;		Anaemia
	Repeat cycle every 21 days		

AE=adverse events.

Adapted with permission from Schiller *et al* (2002). Copyright © 2002 Massachusetts Medical Society. All rights reserved.

) (Schiller *et al*, 2002) and pulmonary adverse events (AEs). Furthermore, debilitating pulmonary and general symptoms are of central concern to patients with advanced NSCLC and lead to severe reductions in quality of life (QoL). The quest is for more effective therapeutic strategies for NSCLC that carry fewer AEs and that balance the clinical benefit gained, including symptom relief, against those AEs.

## ADVANCES IN THE CHEMOTHERAPY OF NSCLC

### The new chemotherapy regimens

Over the past decade, older chemotherapy regimens have been replaced by a number of new chemotherapy agents for the treatment of NSCLC, including the taxanes (paclitaxel and docetaxel), gemcitabine and vinorelbine. Vinorelbine in combination with cisplatin was the first novel combination regimen to produce a statistically significant survival advantage over a standard regimen (vindesine plus cisplatin) (Le Chevalier *et al*, 1994). Subsequently, in a randomised phase III trial, the Southwest Oncology Group confirmed the efficacy of vinorelbine plus cisplatin for patients with metastatic NSCLC (Wozniak *et al*, 1998). Several studies have been performed to compare recent doublet regimens.

A randomised phase III trial compared paclitaxel plus carboplatin with vinorelbine plus cisplatin in patients with metastatic NSCLC (Kelly *et al*, 2001). No difference in efficacy was observed between the two treatment arms, with objective response rates of 25 and 28%, median survival times of 8 months in both arms and 1-year survival rates of 38 and 36%, respectively. Less toxicity was observed with the paclitaxel plus carboplatin regimen.

**Figure 1:**
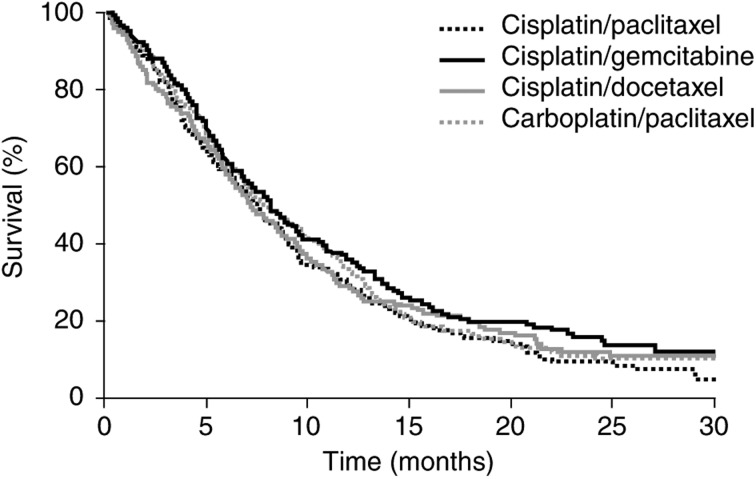
Survival of patients with stage IV NSCLC receiving four different platinum-based combination chemotherapy regimens (Schiller *et al*, 2002). Reprinted with permission from: Schiller *et al* (2002). Copyright © 2002 Massachusetts Medical Society. All rights reserved.

The Eastern Cooperative Oncology Group (ECOG) reported results from a randomised trial in patients with advanced NSCLC that compared the efficacy of a reference regimen of cisplatin and paclitaxel with three experimental regimens: cisplatin and gemcitabine, cisplatin and docetaxel, and carboplatin and paclitaxel (Schiller *et al*, 2002). The response rate and survival did not differ significantly between patients assigned to receive cisplatin and paclitaxel and those assigned to receive any of the three experimental regimens (Figure 1

) (Schiller *et al*, 2002). However, there was a suggestion that time to progression was more favourable with the gemcitabine combination. Treatment with cisplatin and gemcitabine was more likely to cause grade 3, 4 or 5 renal toxicity, while the combination of carboplatin and paclitaxel was considered, comparatively, to be the most tolerable.

Results from a randomised phase III trial of docetaxel plus platinum regimens *vs* vinorelbine plus cisplatin for first-line therapy of advanced NSCLC were recently reported (Fossella *et al*, 2003). A more favourable overall response and survival rate was observed in patients treated with a docetaxel regimen than the vinorelbine combination: overall response rates were 31.6 and 24.5% for patients treated with docetaxel and vinorelbine, respectively (*P*=0.029); median survival was 11.3 months and 10.1 months, respectively. Compared with vinorelbine plus cisplatin, the docetaxel regimen was better tolerated and consistently improved QoL.

Three-drug combinations might also be an option for treatment of NSCLC. However, at least two randomised trials found no benefit for chemotherapy triplets over doublets (Pirker, 2002) and, as toxicity is likely to be high, there is no justification for increasing the number of chemotherapy drugs in a treatment regimen beyond two unless the benefit : risk ratio has been demonstrated to be favourable.

### Cisplatin or carboplatin regimens?

A number of first-line chemotherapy options are advocated in treatment guidelines and/or by various clinical investigators for the treatment of patients with advanced NSCLC. Platinum-based chemotherapy has clearly demonstrated efficacy in patients with advanced NSCLC and there is increasing interest in the use of nonplatinum chemotherapy regimens. Regimens such as cisplatin or carboplatin combined with paclitaxel, vinorelbine, gemcitabine, docetaxel or irinotecan are among the platinum-based combinations currently used in clinical practice. The particular combinations employed may vary between institutions and geographical regions and, although a number of studies have compared cisplatin- with carboplatin-based regimens, no definitive evidence suggests either agent to be better. An early ECOG study compared three cisplatin-based regimens with single-agent carboplatin, and the results demonstrated that carboplatin-treated patients had better overall survival and less toxicity than cisplatin-based regimens (Bonomi *et al*, 1989). Likewise, a randomised phase III study that compared cisplatin and carboplatin in combination with vindesine and mitomycin C indicated a survival advantage for the carboplatin regimen (Jelic *et al*, 2001). In contrast, a paclitaxel/cisplatin regimen was better than a carboplatin regimen (Rosell *et al*, 2002) and no significant differences in survival were apparent in a further trial comparing cisplatin and etoposide with carboplatin and etoposide by the European Organisation for Research and Treatment of Cancer (Klastersky *et al*, 1990).

### Chemotherapy in the elderly and poor performance status (PS) patients

Approximately one-third of all patients with NSCLC are >70 years of age and, although these patients are likely to have an increased risk of comorbid conditions and impaired organ function, most studies have suggested that age alone should not be a factor in the decision to treat patients with chemotherapy. A retrospective study of patients treated for advanced NSCLC found no major differences between patients older or younger than 65 years of age (Giovanazzi-Bannon *et al*, 1994), and an analysis of age as a risk factor in chemotherapy trials found that the response, toxicity and survival rates of elderly patients were similar to those of younger patients (Langer *et al*, 2002).

Two important phase III randomised studies of chemotherapy in the elderly have been reported (Fossella *et al*, 2000; Gridelli, 2001). The Elderly Lung Cancer Vinorelbine Italian Study group performed a multicentre, randomised trial of single-agent vinorelbine as a first-line agent in elderly patients with advanced NSCLC. Results demonstrated that the addition of vinorelbine to best supportive care significantly prolonged survival in this group of patients (Gridelli, 2001). The median survival for best supportive care plus vinorelbine was 28 weeks compared with 21 weeks for best supportive care alone. This benefit was achieved at the cost of some drug-related toxicity, reflected in lower scores on QoL subscales that were directly related to drug toxicity (e.g. nausea and constipation). However, patients who received vinorelbine scored better than control patients on overall health status and QoL, and suffered less from the lung cancer symptoms of dyspnoea, cough and haemoptysis.

More recently, the Multicentre Italian Lung cancer in the Elderly Study randomised elderly patients with advanced NSCLC to vinorelbine, gemcitabine or vinorelbine plus gemcitabine (Gridelli *et al*, 2003). Results demonstrated that the combination of gemcitabine plus vinorelbine in this patient population does not improve survival or QoL compared with single-agent chemotherapy with vinorelbine or gemcitabine. The combination treatment was more toxic than the agents administered alone.

Overall, the studies in elderly patients with NSCLC demonstrate that chemotherapy regimens can lead to an improvement in survival with a better QoL. However, careful attention should be paid to the toxicity of treatment regimens and the coexisting comorbidities in this patient population.

For PS 2 patients, the benefit of chemotherapy remains to be conclusively demonstrated. A survival advantage in this group of patients was demonstrated for carboplatin and paclitaxel compared with single-agent paclitaxel in a phase III randomised trial (Lilenbaum *et al*, 2002). The median survival was 4.7 months for the combination of paclitaxel and carboplatin and 2.4 months for paclitaxel alone. At 1 year, survival rates were 18% for the combination compared with 10% for paclitaxel alone. The outcome of patients with PS 2 was also reported in an ECOG study that compared paclitaxel and cisplatin with three newer chemotherapy doublets in the treatment of patients with advanced NSCLC (Sweeney *et al*, 2001). In this study, patients with PS 2 experienced a large number of AEs and overall poor survival. The overall median survival of all 68 patients who enrolled was 4.1 months. A comparison with patients with a PS of 0–1 suggests that the shorter survival in patients with a PS of 2 was related to tumour progression rather than treatment. Alternative strategies specifically tailored for this group of patients need to be further explored.

### Advances in second-line chemotherapy

With increased use of chemotherapy, the number of patients seeking second-line therapy after relapse following first-line treatment has increased. Available treatments for second-line therapy are limited and patients with tumour progression at this stage generally have very poor prognoses.

The TAX 320 NSCLC Study Group performed a phase III trial in which patients with advanced NSCLC who had previously failed platinum-containing chemotherapy were randomised to 75 or 100 mg m^−2^ docetaxel or a reference regimen of vinorelbine or ifosfamide (Fossella *et al*, 2000). Overall response rates were 6.7 and 10.8% with 75 and 100 mg m^−2^ docetaxel, respectively, each significantly higher than the 0.8% response with vinorelbine or ifosfamide. Overall survival did not differ significantly between the three groups, although the 1-year survival was significantly greater with 75 mg m^−2^ docetaxel than the control treatment.

In another trial, patients with NSCLC were randomised to either docetaxel or best supportive care (Shepherd *et al*, 2000). Docetaxel was initially administered at 100 mg m^−2^ every 21 days, but was later reduced to 75 mg m^−2^ every 21 days due to an unacceptable toxic death rate at the higher dose. Despite low overall response rates, patients treated with docetaxel survived longer. The overall response rate for docetaxel, based on 84 patients with measurable lesions, was 7.1%. The median survival was 7.0 months for patients receiving docetaxel compared with 4.6 months for patients receiving best supportive care. Quality-of-life analysis demonstrated less worsening of PS and less use of tumour-related medications for docetaxel-treated patients.

Although docetaxel appears to demonstrate the most consistent efficacy as a second-line treatment and has recently been approved for first-line treatment when combined with cisplatin, it is associated with a high incidence of haematological toxicity. An alternative chemotherapy treatment option is the experimental agent pemetrexed, a new antifolate. In a phase III study in previously treated patients with recurrent NSCLC, pemetrexed (used with vitamin supplements) had a more favourable haematological toxicity profile when compared with docetaxel (Hanna *et al*, 2003); patients experienced less severe neutropenia, fewer hospitalisations and less need for granulocyte colony-stimulating factor/granulocyte–macrophage colony-stimulating factor support. Pemetrexed therapy, while less toxic, was still associated with haematological toxicity, hospital admissions and haematological growth factor support, and did not improve overall outcome compared with docetaxel; hence, there is still an unmet need for efficacious, well-tolerated treatment options.

### The therapeutic ratios of chemotherapy regimens

In making decisions for treatment regimens, it is necessary to consider the overall therapeutic index, that is, not just the response rate or survival but also the toxicity of treatment and the subsequent outcomes. Cytotoxic chemotherapy regimens are often associated with significant toxicity, which has a detrimental effect on a patient's QoL. Furthermore, such toxicity may result in a patient opting not to receive chemotherapy. Indeed, in a recent study assessing the treatment preferences of patients with lung cancer, only 22% said they would choose chemotherapy over best supportive care for a 3-month improvement in survival, while the majority (68%) would choose chemotherapy if it substantially reduced symptoms without an improvement in survival (Silvestri *et al*, 1998).

### The unmet need for new paradigms of treatment for NSCLC

**Figure 2:**
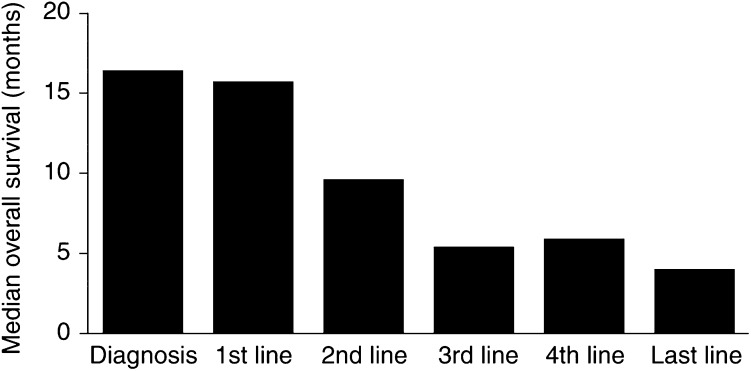
Median overall survival time for each line of treatment (Massarelli *et al*, 2003). Reprinted from: Massarelli *et al* (2003). Copyright (2003), with permission from Elsevier.

Chemotherapy treatment and supportive care to manage some unwanted side effects has progressed in the past decade. Indeed, the new doublet treatment regimens provide advantages for patients and some improvement in survival and QoL over the older chemotherapy regimens. Despite these advances in treatment, improvements in the survival of patients with NSCLC in recent years have been modest. For patients with advanced disease with good enough PS to tolerate and thus receive third- or fourth-line chemotherapy, a retrospective analysis reported the median overall survival from the start of the last treatment as 4 months (Figure 2

) (Massarelli *et al*, 2003).

Clinical data over the past decade show that there is unlikely to be further significant improvement in outcome using conventional treatment and, as there are few options for patients who have relapsed following chemotherapy, there is an unmet need for the treatment of NSCLC.

The trend of not receiving any chemotherapy with curative intent is especially common among elderly patients. In a study of the management of 6300 Medicare patients aged >65 years who were diagnosed with stage IV NSCLC, the authors found that only 21.5% received chemotherapy for metastatic disease (Earle *et al*, 2000). They noted that despite the fact that all patients had the same health insurance cover, the use of chemotherapy was far from consistent for all beneficiaries. In this cohort, younger patients and those with few comorbid conditions were more likely to receive chemotherapy but several nonmedical factors, such as nonblack race, higher socioeconomic status, treatment in a teaching hospital and living in certain urban areas, also significantly increased a patient's likelihood of receiving chemotherapy (Earle *et al*, 2000). Scepticism towards treatment was also common among Canadian physicians; only 20% thought that treating advanced NSCLC with chemotherapy was worthwhile (Raby *et al*, 1995). In the UK, only 11% of clinicians recommended chemotherapy for appropriate elderly patients, whereas 26% advised therapy if the patient was <50 years of age (Crook *et al*, 1997). New modalities of therapy may improve these numbers and increase the percentage of treated patients.

## GEFITINIB: A NOVEL TARGETED THERAPY FOR NSCLC

### Epidermal growth factor receptor (EGFR)

The EGFR is expressed in a wide range of solid tumours and is associated with a poor prognosis in several malignancies. Indeed, evidence for a role for the EGFR in the inhibition and pathogenesis of various cancers has led to the development of agents that target this receptor. Those most advanced in development are the EGFR tyrosine kinase inhibitors (EGFR-TKIs) gefitinib (‘Iressa’) and erlotinib (‘Tarceva’), and the monoclonal antibody cetuximab (‘Erbitux’).

### Clinical trials with gefitinib

Gefitinib is the EGFR-TKI with the most extensive clinical experience, particularly in NSCLC. Based on data from two pivotal phase II trials, IDEAL (‘Iressa’ Dose Evaluation in Advanced Lung cancer) 1 and 2 (Fukuoka *et al*, 2003a; Kris *et al*, 2003), gefitinib 250 mg once daily was recently approved for use in patients with previously treated advanced NSCLC in Japan, the USA and other countries. Patients in both studies had been pretreated and, while all patients had a poor prognosis, patients in IDEAL 1 had a better prognosis than those in IDEAL 2. In IDEAL 1, patients had received one or two prior chemotherapy regimens (at least one platinum based), whereas in IDEAL 2 all patients had received two or more prior chemotherapy regimens (which must have included platinum and docetaxel). All had received their last dose of chemotherapy within the previous 90 days and had recurrent disease or were unable to tolerate further chemotherapy, usually as a result of chemotherapy-associated neuropathy. Furthermore, all patients in IDEAL 2 and 66.7% of patients in IDEAL 1 were symptomatic at trial entry. At the recommended dose of 250 mg day^−1^, the objective tumour response rates were 18.4 and 11.8% in IDEAL 1 and 2, respectively. Objective responses observed in patients with large or bulky measurable tumours and in patients with extensive, nonmeasurable disease were also durable; median durations of response at 250 mg day^−1^ were 13.0 and 7.0 months in IDEAL 1 and 2, respectively (Fukuoka *et al*, 2003b). Survival data were encouraging and disease control rates (response plus stable disease) showed that almost half of the patients treated with gefitinib in both trials benefited from treatment: median survival rates at 250 mg day^−1^ were 7.6 and 6.5 months in IDEAL 1 and 2, respectively; disease control rates at 250 mg day^−1^ were 54.4 and 42.2%, respectively. In both IDEAL 1 and 2, disease-related symptoms improved significantly for many patients. At the recommended dose of 250 mg day^−1^ gefitinib, 40.3 and 43.1% of symptomatic patients in IDEAL 1 and 2, respectively, experienced improvement in their disease-related symptoms that lasted for at least 1 month. Symptom relief was also rapid: the median time to onset of improvement was 8 days in IDEAL 1 and 10 days in IDEAL 2 (the times of the first postbaseline assessment for each study).

Results of preliminary studies suggested that first-line combination therapy with gefitinib and platinum-based chemotherapy is feasible and support further investigation of these combinations in NSCLC. However, in the phase III combination trials INTACT (‘Iressa’ NSCLC Trial Assessing Combination Treatment) 1 and 2, which involved >2100 chemonaive patients with advanced NSCLC, gefitinib provided no additional survival benefit in the first-line setting. Nevertheless, results confirmed the safety role of gefitinib in a placebo-controlled setting (Johnson *et al*, 2002): there was no worsening of chemotherapy-associated toxicities and the observed side effects of skin rash, acne and diarrhoea were as predicted from the IDEAL trials. In light of monotherapy activity, the reasons for the lack of increased activity in combination therapy are yet to be determined, although current studies are trying to analyse retrospectively the biological profile of responders *vs* nonresponders.

## ADVANCES IN THE TREATMENT OF NSCLC IN JAPAN

### The Japanese incidence rates of NSCLC

Lung cancer remains the leading cause of cancer-related death in Japan despite efforts to promote early detection and surgery. Statistics from the Japanese Ministry of Health, Labour and Welfare show approximately 55 000 deaths from lung cancer yearly in Japan and predict an 80% increase in incidence over the next 15 years.

### Types of treatment for lung cancer

Globally, surgery is the treatment of choice for patients with stage I and II NSCLC and for patients with advanced disease, the principal forms of treatment are radiation therapy, chemotherapy, surgery and a combination of these options. In Japan, in contrast to the rest of the world, surgery is the primary treatment option offered to those with advanced disease. UFT, a combination of tegafur and uracil, is also widely available in Japan, where researchers have demonstrated the utility of this agent as adjuvant therapy after surgical resection, whether alone or in combination with cisplatin and vindesine (Langer, 1999). Further research is required to determine whether this agent will have a role in treatment of NSCLC outside Japan.

### Preferred chemotherapy regimens in Japan

**Figure 3:**
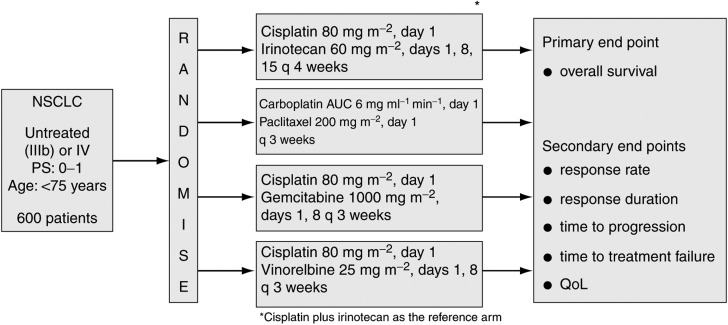
Four-Arm Cooperative Study for advanced NSCLC in Japan: trial design.

In Japan, platinum-based regimens are the preferred standard chemotherapy for NSCLC at present. However, chemotherapy for NSCLC in Japan is controversial because the differences in the efficacies of combination chemotherapies, including new agents such as irinotecan, paclitaxel and vinorelbine, have not been recognised in randomised controlled trials. The Four-Arm Cooperative Study for advanced NSCLC is an ongoing postmarketing clinical trial in Japan that was designed to compare three platinum-based combination regimens (carboplatin plus paclitaxel, cisplatin plus gemcitabine and cisplatin plus vinorelbine) with cisplatin plus irinotecan as the reference arm (Ohe *et al*, 2003) (Figure 3

). To date, similar response rates have been observed in all four treatment arms. Toxicity was also a feature of each treatment arm: thrombocytopenia and diarrhoea were very common. There is no ‘Gold Standard’ therapy for the treatment of NSCLC in Japan and the development of new agents, particularly molecular-target-based drugs such as gefitinib, is necessary to further improve therapeutic results in lung cancer.

Approximately 37 000 patients have received gefitinib in Japan and, up to January 2003, 84 patients had received gefitinib in clinical practice at the Tokyo Medical University (Table 2

**Table 2:** Tokyo Medical University experiences with gefitinib: patient demography

	**Gefitinib, 250 mg day^−1^**
No. patients treated	84
Mean (range) age, years	65.5 (35–87)
Male : female, *n*	44 : 40
	
*Performance status, n*	
0–1	75
2	9
	
*Tumour histology, n*	
Adenocarcinoma	68
Nonadenocarcinoma	16
	
*Disease stage*	
IV	32
III a/b	10/23
II a/b	1/0
Postoperative	18
	
*Previous cancer treatment, n*	
0 prior regimens	1
1 prior regimen	35
⩾2 prior regimens	48

). Overall, the partial response rate was 15.5% and stable disease rate was 45.2%. In this difficult-to-treat patient population, the median time to disease progression was 4 months. Gefitinib was generally well tolerated: grade 3/4 AEs included one pulmonary, one skin and one hepatic event. Individual patients also experienced improvement in QoL. For example, a patient with NSCLC liver metastases was able to perform activities not previously possible following the administration of gefitinib. Furthermore, an improvement in liver function was observed.

The incidence of interstitial lung disease (ILD) in patients treated with gefitinib in Japan was recently reported as being 2% (Forsythe and Faulkner, 2003). However, the reported incidence of ILD in >80 000 patients who have received gefitinib worldwide is approximately 1%, and it is 0.3% in a worldwide compassionate-use programme in >38 000 patients, in which investigators must submit safety data (Forsythe and Faulkner, 2003). Although vigilance for pulmonary toxicity is mandatory, in this area of high unmet clinical need, the benefits of gefitinib outweigh the risks in patients for whom no other proven effective treatment option exists.

The antitumour activity of gefitinib, together with its favourable tolerability profile and oral bioavailability in patients with advanced NSCLC who have previously received treatment with cytotoxic chemotherapy, has led to the development of a programme of clinical trials to define the further potential of gefitinib in NSCLC. In Japan, planned or ongoing trials will assess the effect of gefitinib as monotherapy and in combination with radiotherapy and chemotherapy. A translational research project is also ongoing. The outcome of these and other trials may lead to opportunities to change the current treatment strategies for NSCLC.

### Future therapies for NSCLC

Despite recent advances in treatment of NSCLC with chemotherapeutic regimens, the prognosis for this patient population remains poor. Molecular targets have recently been identified as a result of advances in molecular biology; subsequently, many novel categories of anticancer drugs have been developed. One example of this approach is a better understanding of growth signalling pathways. The EGF pathway contributes to the malignant potential of NSCLC and several compounds have been developed to selectively block this pathway, for example, the EGFR-TKIs gefitinib and erlotinib and the monoclonal antibody cetuximab. Most advanced in research are the TKIs, which have demonstrated similar efficacy and safety profiles. Gefitinib is the agent with the most extensive clinical experience and recently it was approved for use as a monotherapy in patients with previously treated advanced NSCLC in Japan, the USA and other countries. Thus, gefitinib is already providing responses and disease stabilisation in patients with NSCLC and no other treatment options.

Further research is required to better understand the role of the EGFR in NSCLC since its significance in individual tumours has not yet been fully identified. Determining the specific cases in which the EGFR plays a pivotal function in carcinogenesis may help to recognise those patients who may benefit from therapy with EGFR inhibitors. Several research directions address this issue: animal studies have demonstrated that EGFR-TKIs have a better antitumour effect when both the tumour and the tumour-associated endothelial cells express the target receptor (Baker *et al*, 2002); and human NSCLC tumour analyses have shown that coexpression of both the EGFR and HER2 affected survival more than either of the receptors alone (Brabender *et al*, 2001; Onn *et al*, 2004). In addition to studies of the role of the EGFR in NSCLC, investigation of different combination therapies is still required and the sequencing of biological compounds with conventional chemotherapy or radiotherapy may provide evidence for directing future studies. Indeed, the molecular diversity of tumours may ultimately necessitate a combination of biological agents to maximise therapeutic effects.
